# Tax-funded social health insurance: an analysis of revenue sources, Hungary

**DOI:** 10.2471/BLT.18.218982

**Published:** 2019-02-28

**Authors:** Szabolcs Szigeti, Tamas Evetovits, Joseph Kutzin, Péter Gaál

**Affiliations:** a Country Office in Hungary, World Health Organization Regional Office for Europe, Budapest, Hungary.; b Division of Health Systems and Public Health, World Health Organization Regional Office for Europe, Copenhagen, Denmark.; c Department of Health Systems Governance and Financing, World Health Organization, Geneva, Switzerland.; d Health Services Management Training Centre, Semmelweis University, H-1125, Kútvölgyi út 2, Budapest, Hungary.

## Abstract

Health financing is a complex health system function, which cannot be analysed accurately without tracking each step of the flow of funds separately. We analysed the revenue mix of the Hungarian health insurance fund from 1994 to 2015 and discuss the policy implications of our findings. We used the System of Health Accounts published in 2000 and the revised version of 2011, which introduced separate classifications for the sources of health expenditure. Based on the 2000 version, health insurance contributions were the main source of public funding in Hungary. According to the 2011 version, nearly 70% of health insurance fund revenues came from government tax transfers in 2015, illustrating the striking difference in how revenues and expenditures are reported using this version. Use of the 2011 version will better inform national policy-making and international comparisons and facilitate documentation and analysis of how countries have adapted their revenue mix to changing macroeconomic circumstances. The finding that Hungary has a predominantly tax-funded social health insurance system suggests that traditional understanding and description of health-financing models are no longer adequate and may limit consideration of potential resource-generation options. Hungary is also a good example of how separating revenue generation and pooling broadens policy options to tackle gaps in social health insurance coverage, although the government did not act on these due to the lack of a consistent health-financing strategy. The findings may be particularly relevant for low- and middle-income countries that are trying to expand social health insurance coverage despite limited formal employment.

## Résumé

Le financement de la santé est une fonction complexe du système de santé, qui ne peut pas être précisément analysée sans étudier séparément chaque étape du flux de fonds. Dans cet article, nous analysons le mix de recettes du fonds d'assurance maladie hongrois de 1994 à 2015 et nous évoquons les implications de nos constatations sur la définition des politiques. Nous avons utilisé le Système des Comptes de la Santé publié en 2000 ainsi que sa version révisée de 2011, qui a introduit des classifications différentes pour les sources des dépenses de santé. En se fondant sur la version de 2000, ce sont les cotisations d'assurance maladie qui ont constitué la principale source de financement public en Hongrie. Mais d'après la version de 2011, près de 70% des recettes constitutives des fonds de l'assurance maladie sont provenues de transferts fiscaux gouvernementaux en 2015, ce qui illustre la différence flagrante dans la manière d'enregistrer les recettes et les dépenses proposée par cette version révisée. L'utilisation de la version de 2011 permettra de mieux informer le processus d'élaboration des politiques nationales, de faciliter les comparaisons internationales ainsi que de mieux documenter et analyser la manière dont les pays adaptent leur mix de recettes face à l'évolution des circonstances macroéconomiques. Le fait que le système d'assurance maladie sociale de Hongrie s'avère principalement financé par l'impôt montre que la compréhension et la description habituelles des modèles de financement de la santé ne sont plus adaptées et que cela peut même entraver la considération d'autres options envisageables pour générer des recettes. La Hongrie est également un bon exemple illustrant comment le fait de séparer la génération des recettes et la mise en commun des fonds élargit les options politiques pour réduire les déficiences dans la couverture de l'assurance maladie sociale, même si le gouvernement n'a pas agi sur ce point, faute de stratégie de financement de la santé cohérente en la matière. Ces constatations peuvent être particulièrement utiles pour les pays à revenu faible et intermédiaire qui essayent d'étendre la couverture de leur assurance maladie sociale malgré un niveau d'emploi limité dans le secteur formel.

## Resumen

La financiación de la salud es una función compleja del sistema sanitario que no puede analizarse con precisión si no se hace un seguimiento independiente de cada paso del flujo de fondos. Se ha analizado la combinación de ingresos de la caja húngara de seguros médicos de 1994 a 2015 y se han discutido las implicaciones políticas de los resultados. Se ha usado el Sistema de Cuentas de Salud publicado en 2000 y la versión revisada de 2011, que introdujo las clasificaciones separadas para las fuentes de gasto en salud. Según la versión de 2000, las cotizaciones al seguro de enfermedad eran la principal fuente de financiación pública en Hungría. Según la versión de 2011, casi el 70 % de los ingresos de la caja de seguros médicos procedían de las transferencias de impuestos del gobierno en 2015, lo que ilustra la sorprendente diferencia en la forma en que se informan los ingresos y los gastos utilizando esta versión. El uso de la versión de 2011 servirá de base para la formulación de políticas nacionales y comparaciones internacionales y facilitará la documentación y el análisis de cómo los países han adaptado su combinación de ingresos a las cambiantes circunstancias macroeconómicas. La conclusión de que Hungría tiene un sistema de seguridad social financiada principalmente por los impuestos sugiere que la comprensión y la descripción tradicionales de los modelos de financiación sanitaria ya no son adecuados y limitan la consideración de las posibles opciones de generación de recursos. Hungría es también un buen ejemplo de cómo la separación entre la generación de ingresos y la puesta en común amplía las opciones políticas para abordar las brechas en la cobertura de la seguridad social, aunque el gobierno no haya actuado al respecto debido a la falta de una estrategia coherente de financiación sanitaria. Las conclusiones pueden ser particularmente pertinentes para los países de ingresos bajos y medianos que estén tratando de ampliar la cobertura de la seguridad social a pesar de la limitación del empleo formal.

## ملخص

التمويل الصحي هو نظام صحي معقد، لا يمكن تحليله بدقة دون تتبع كل خطوة من خطوات تدفق الأموال كل على حدة. قمنا بتحليل مزيج الإيرادات من صندوق التأمين الصحي الهنغاري من عام 1994 إلى عام 2015، وناقشنا الآثار السياسية لنتائجنا. استخدمنا نظاماً للحسابات الصحية، تم نشره في عام 2000، وصدرت النسخة المنقحة منه عام 2011، والذي أدخل تصنيفات منفصلة لمصادر الإنفاق الصحي. واستناداً إلى نسخة عام 2000، كانت مساهمات التأمين الصحي هي المصدر الرئيسي للتمويل العام في هنغاريا. وفقاً لنسخة 2011، جاء ما يقرب من 70٪ من إيرادات صندوق التأمين الصحي من تحويلات الضرائب الحكومية في عام 2015، وهو ما يوضح الفارق الشاسع في كيفية الإبلاغ عن الإيرادات والنفقات باستخدام هذه النسخة. إن استخدام نسخة 2011 سيعمل على تحسين صياغة السياسات الوطنية والمقارنات الدولية وتسهيل التوثيق والتحليل لكيفية تكييف البلدان لمزيج عائداتها مع تغير الظروف الاقتصادية الكلية. إن النتيجة التي مؤداها وجود نظام تأمين صحي اجتماعي بتمويل من الضرائب في المقام الأول، تشير إلى أن الفهم والتوصيف التقليدي لنماذج التمويل الصحي لم يعودا كافيين، وقد يحدان من النظر في الخيارات المحتملة لتوليد الموارد. هنغاريا هي أيضا مثال جيد على كيفية فصل توليد وتجميع الإيرادات، يؤدي إلى توسيع خيارات السياسة لمعالجة الثغرات في تغطية التأمين الصحي الاجتماعي، على الرغم من أن الحكومة لم تتخذ اللازم بهذا الصدد نظرا لعدم وجود استراتيجية متسقة للتمويل الصحي. قد تكون النتائج ذات صلة خاصة بالبلدان ذات الدخل المنخفض والدخل المتوسطة، التي تحاول توسيع تغطية التأمين الصحي الاجتماعي على الرغم من محدودية العمالة الرسمية.

## 摘要

卫生筹资是一项复杂的卫生系统功能，如果不对资金流动的每一步进行单独追踪，我们就无法对其进行准确分析。本文对 1994 年至 2015 年匈牙利医疗保险基金的收入构成进行了分析，并讨论了调查结果的政策意涵。本文使用《卫生核算体系 2000》以及《卫生核算体系 2011》（修订版），其中对卫生支出来源进行了单独分类。根据 2000 年的版本，匈牙利公共资金的主要来源是医疗保险缴费。根据 2011 年的修订版，近 70% 的医疗保险基金收入来自 2015 年政府的税收转移，该研究表明使用此版本报告收入和支出的方式存在显著差异。使用 2011 年的修订版更有利于国家政策的制定和国际间的比较，并在记录和分析各国如何调整其收入构成以适应不断变化的宏观经济环境方面起到了促进作用。匈牙利的社会医疗保险体系主要来自于税收资金，这一调查发现表明，对卫生筹资模式的传统理解和阐释已不再适用，并且可能会限制对潜在资源生成选项的考量。尽管由于缺乏一致的卫生筹资战略，政府并未对此采取行动，但匈牙利在如何区分收入的产生和汇总并扩大政策选择范围，以缩小社会医疗保险覆盖范围的差距方面起了很好的示范作用。调查结果可能尤其适用于低收入和中等收入国家，尽管这些国家在正式就业方面受限，但其正在努力扩大社会医疗保险的覆盖范围。

## Резюме

Финансирование здравоохранения — сложная функция системы здравоохранения, которую невозможно точно проанализировать без отслеживания каждого из этапов потоков финансирования в отдельности. Авторы проанализировали структуру дохода венгерских фондов медицинского страхования за период с 1994 по 2015 год и рассматривают практические выводы на основе полученных результатов. Использовалась система счетов сектора здравоохранения, опубликованная в 2000 году, и пересмотренная версия 2011 года, в которой внедрены отдельные классификации для источников расходов на здравоохранение. По данным версии 2000 года, взносы на медицинское страхование служили основным источником государственного финансирования в Венгрии. Согласно версии 2011 года, в 2015 году около 70% дохода фондов медицинского страхования составили перечисления из налоговых поступлений, что свидетельствует о существенной разнице в том, каким образом ведется учет дохода и расходов в данной версии. Версия 2011 года предоставляет больше информации для разработки национальной политики и осуществления международного сопоставления, а также облегчает процесс документирования и анализа данных о том, как страны адаптируют структуру дохода применительно к изменяющимся макроэкономическим условиям. Полученные данные, свидетельствующие о том, что система медико-социального страхования в Венгрии преимущественно финансируется из налоговых поступлений, позволяют предположить, что традиционное понимание и описание моделей финансирования в здравоохранении более не соответствуют реальности и могут ограничивать рассмотрение потенциальных ресурсов поступления доходов. Венгрия также является хорошим примером того, как разделение поступления доходов и объединения средств увеличивает количество альтернативных стратегий по ликвидации пробелов в покрытии медико-социального страхования, хотя правительство не предпринимало никаких действий для решения данной проблемы по причине отсутствия последовательной стратегии финансирования здравоохранения. Полученные результаты могут оказаться важными для стран с низким и средним уровнем дохода, желающих расширить сферу действия медико-социального страхования, несмотря на ограниченность официальной занятости.

## Introduction

**Fig. 1:**
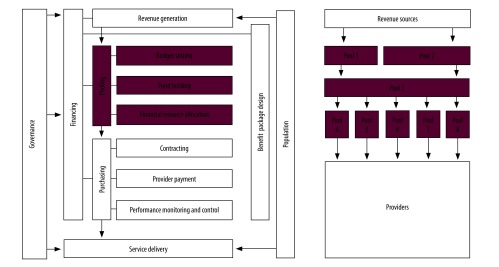
Subfunctions of the health-financing function and its interconnectedness with other health system functions

Note: Arrows represent the flow of funds.

Source: Adapted from Kutzin, 2010.^1^

Health financing is a key health system function. Such financing can be divided into several subfunctions according to the way the money flows in the health system: from households, which are the ultimate source of health revenues, through financial intermediaries, which manage budgets, to health care organizations, which provide services to patients (Fig. 1). Accordingly, revenue generation can be separate from pooling of funds, and pooling from purchasing. Using the case of Hungary, we argue that these distinctions are important for policy-making as they allow a wider range of policy options to be considered to improve health system performance. As a starting point for analysis, financial resources should be tracked using a standard method that makes the documentation of the flow of funds in the health system detailed enough to distinguish the financing subfunctions from each other and allows comparison between countries and over time.

The System of Health Accounts is a joint effort of the World Health Organization (WHO), the statistical office of the European Union, and the Organisation for Economic Co-operation and Development (OECD) to establish a standard framework for tracking resources to describe and analyse health-financing arrangements in member countries.^2^ The system classifies health expenditures into well-defined categories of the various health system dimensions, such as financing schemes, providers and services. The first version of the System of Health Accounts, introduced in 2000 by OECD,^3^ was followed by a revised version in 2011.^2^ One of the most important improvements of the new version is its ability to distinguish the sources of revenue from pooled funds managed by financing agents. The 2000 version classified data on the structure and composition of revenue sources according to financing agent, which meant, for instance, that all the revenues of a social health insurance fund were considered health insurance contributions, even if some came from the central government budget to cover non-contributing groups.^3^ The 2011 version introduced two new classifications (Box 1), health-financing schemes and revenues of health-financing schemes, which enable analysis of revenue sources separately from that of pooling and purchasing arrangements for a particular scheme.

Box 1 **Revenue categories and health-financing schemes based on the 2011 version of the System of Health Accounts^2^**

The health financing schemes of the International Classification for Health Accounts are divided into eight main categories (ICHA-HF):

- HF.1.1 Government financing schemes
- HF.1.2 Compulsory contributory health insurance schemes
- HF.1.3. Compulsory medical savings accounts (CMSA)
- HF.2.1 Voluntary health insurance schemes
- HF.2.2 Non-profit institutions financing schemes
- HF.2.3 Enterprise financing schemes (other than employer-based insurance)
- HF.3 Household out-of-pocket expenditure
- HF.4 Rest of the world financing schemes

The revenues of these health-financing schemes (ICHA-FS) are classified into seven main categories, which are further divided into subcategories:

- FS.1 Transfers from government domestic revenue
- FS.1.1 Internal transfers and grants
- FS.1.2 Transfers by government on behalf of specific groups
- FS.1.3 Subsidies
- FS.1.4 Other transfers from government domestic revenue
- FS.2 Transfers distributed by government from foreign origin
- FS.3 Social insurance contributions
- FS.3.1 Social insurance contributions from employees
- FS.3.2 Social insurance contributions from employers
- FS.3.3 Social insurance contributions from self-employed
- FS.3.4 Other social insurance contributions
- FS.4 Compulsory prepayment (other than FS.3)
- FS.5 Voluntary prepayment
- FS.6 Other domestic revenues not elsewhere classified
- FS.7 Direct foreign transfers

The European debt crisis that started in 2009 brought the sustainability of health financing to the forefront of policy debates. In Hungary, the government spoke about the need to balance the budget of the health insurance fund,^4^ and internationally it was thought that sustainability could be enhanced by increasing reliance on general taxation.^5^^,^^6^ WHO suggested alternative financing sources beside social health insurance contributions and other types of wage-based revenue sources, which increase the cost of labour, to mitigate their implied adverse effects on employment and economic growth.^7^ Hungary provides a useful illustration of this alternative financing as successive governments have tried to boost employment by reducing the social insurance contribution rate and compensated for this reduction by increasing tax financing of the health insurance fund.^8^^,^^9^

Until 2017, OECD and WHO published comparable data based on the 2000 version of the System of Health Accounts.^10^^–^^12^ These data, however, cannot show changes over time in the composition of financing sources for the health insurance fund because the 2000 version does not disaggregate revenue sources and classifies all expenditures of the health insurance fund as a health insurance source. To reveal the sources of health insurance fund expenditures, we therefore used the approach of the 2011 version.^2^

Using the case of Hungary, this paper has two main objectives. First, to analyse the changes in the revenue sources of the health insurance fund using the framework of the 2011 version and demonstrate the importance and feasibility of this type of analysis. Second, to discuss the policy relevance and implications of the findings.

## Hungarian health system

After the collapse of the communist regime in 1990, Hungary replaced the socialist state health system with a social health insurance scheme. Despite some recent changes, this scheme is still the backbone of the health system: tax and social health insurance contribution revenues are channelled into one national pool, the health insurance fund, which is managed by a single payer, the National Health Insurance Fund Administration. The administration contracts almost exclusively with public (central, or local government-owned) providers, which are paid based on the type of service they supply: there is capitation payment in primary care, outpatient specialist services are paid for by fee-for-service, while acute inpatient care is covered by a Hungarian version of diagnosis-related groups. In a diagnosis-related group system, hospital patients are put into groups by diagnosis and treatment and each group has an assigned point value. A more serious disease with expensive treatment has more points than a simple, less costly case. Hospitals are paid according to the number of diagnosis-related group points and not simply the number of patients treated.^8^^,^^13^

Participation in the social health insurance scheme is compulsory; opting out is not permitted. Employers pay a so-called social tax at a fixed percentage, employees pay a social health insurance contribution (currently 7% of gross wage) and the central government covers non-contributing population groups, e.g. pensioners, minors and students. The public benefit package, which is the health services covered by the social health insurance scheme, is comprehensive (with few exclusions) and population coverage was 94.9% in 2017.^8^^,^^14^

In Hungary, concerns about health-financing policy are mainly about the effect of revenue generation on the labour market, the stability and sustainability of public financing, and the high level of out-of-pocket payments.^8^^,^^9^ Gaps in population and service coverage are less of a policy concern in terms of revenue generation. Therefore, we made the mix of revenue sources, in particular taxes and earmarked payroll revenues, the focus of our analysis because of the potential adverse effects of payroll revenues on employment and the challenges of collection in the informal sector.^15^^,^^16^

## Analysis of revenues

We obtained published statistics of the National Health Insurance Fund Administration, the financing agent of the Hungarian social health insurance scheme.^17^^–^^21^ Then, we looked at government acts on reporting the implementation of the budget of the health insurance fund for items that needed clarification or were entirely missing from the yearbooks of the administration. These acts contain a detailed breakdown of the revenues and expenditures of the health insurance fund.^22^^–^^27^ Table 1 presents revenue sources for each budget line based on the published statistics and complemented with data from the acts.

**Table 1:** Revenue sources of the Hungarian health insurance fund, 1995, 2000, 2005, 2008, 2010 and 2015

Categories and items	Revenue (million Hungarian forints) by year					
	1995	2000	2005	2008	2010	2015
**Social health insurance contributions and other contributions**	344 717	653 715	1 104 335	1 028 377	677 734	1 223 992
1. Employer contribution	286 600	371 560	678 392	411 813	159 721	352 166
2. Employee contribution	46 867	81 314	227 707	447 761	431 835	652 182
3. Contribution by special groups (compulsory participation)	1 837	946	3 793	17 085	21 232	29 145
4. Contribution based on voluntary agreement	–	510	650	226	229	333
5. Employer repayment of sick pay	–	13 387	23 165	24 894	18 833	19 607
6. Contribution paid in connection with short-term employment	–	31	707	427	147	200
7. Compensation by the labour market fund for contribution relief in the Start Card programme and other tax transfers from the labour market fund	–	–	–	2 499	1 473	–
8. Contribution for conscripts^a^	–	560	–	–	–	–
9. Special contribution to disability pension for members of the armed forces^a^	–	1 008	1 279	–	–	–
10. Hypothecated health-care tax	–	181 379	164 408	118 968	41 207	166 362
11. Late payment and other fines	9 413	3 020	4 234	4 703	3 058	3 995
**Central budget contributions**	14 782	70 872	66 050	354 385	617 271	564 935
12. Tax transfers for abortion^b^	–	900	1 250	1 500	1 600	–
13. Tax transfers for non-contributing groups	10 400	46 572	0	307 038	611 771	374 224
14. Tax transfers for special health services^b^	4 382	2 900	3 500	3 800	3 900	5 400
15. Tax transfers for maternity pay	–	20 500	61 300	42 047	–	–
16. Tax transfers for disability and rehabilitation benefits	–	–	–	–	–	155 311
17. Tax transfer for unspecified tasks	0	0	0	0	0	30 000
**Other revenues**	59 044	2 635	31 266	59 015	88 273	135 121
18. User charges for abortion	169	334	667	670	605	525
19. Reimbursement of health insurance fund expenditures on accidents and other injuries/damages by the responsible entity (e.g. compulsory third-party liability insurance for motor vehicles)	1 090	840	5 442	6 441	6 176	5 696
20. Other repayments and special revenues	1 059	980	1 923	1 541	1 636	1 419
21. Transfer from the pension insurance fund	56 726	–	–	–	–	–
22. Repayment of pharmaceutical subsidies by pharmaceutical companies	–	–	23 077	38 799	50 936	65 272
23. Reimbursement of health insurance fund expenditures based on international agreements (EU social security coordination and other bilateral agreements)	0	0	93	270	1 146	4 248
24. Repayment of health insurance fund reimbursements by health-care providers	–	481	60	335	294	1 036
25.User charges for patient–doctor encounters and for hospital stay (visit fee, hospital daily user charge)	–	–	–	10 960	–	–
26. Additional tax transfers from the 2007 surplus of the health insurance fund	–	–	–	–	27 481	–
27. Tax on the premium of compulsory third-party liability insurance for motor vehicles	–	–	–	–	–	27 493
28. Public health product tax on unhealthy food	–	–	–	–	–	28 891
29. Tobacco industry health tax	–	–	–	–	–	540
**30. Revenues from asset management**	709	3 885	207	26	12	14
**31. Revenues from administrative fees**	3 663	3 000	2 739	3 382	1 702	1 996
**Total revenues**	**422 915**	**734 108**	**1 204 597**	**1 445 184**	**1 384 992**	**1 926 058**

EU: European Union.

^a^ These are revenue items that are not included in the yearbooks of the National Health Insurance Fund Administration, but are included in the total revenue.

^b^ This item is included in tax transfers for special health services for 1995 and 2015.

Notes: Dashes indicate that the revenue item had not yet been introduced or had been discontinued in that year. Data for 2005 were obtained from the 2010 yearbook of the National Health Insurance Fund Administration^20^ and data for 2008 were obtained from the 2009 yearbook.^19^ One million Hungarian forints are equivalent to 3700 United States dollars (2018 currency rate). The English translation of the various items in the publications does not follow the terminology established in the scientific literature on health-care financing.^28^ Therefore, we revised the original English translation of revenue sources. We also numbered the items.

Data source: National Health Insurance Fund Administration.^17^^–^^21^

We assessed each budget line against the classification of revenues of health-financing schemes defined in the 2011 version of the System of Health Accounts and assigned each to the appropriate revenue category (Table 2). The health accounts manual defines seven main categories of revenues of health-financing schemes (FS.1 to FS.7).^2^ From our perspective, the most important distinction was between government domestic revenues (FS.1) and health insurance contributions (FS.3) because the other five categories constitute only a small fraction of the revenues of the health insurance fund, and social health insurance contributions are assumed to have an adverse effect on employment. However, we first had to consider special revenue items, which belong to one of the other five groups, or which fall outside the health sector and therefore had to be excluded, before looking at whether the remaining budget lines could be classified as a social health insurance contribution (FS.3) or a tax (FS.1). According to the classification of the 2011 version, there are two important features of health insurance contributions: they are employment-based (paid by employers on behalf of their employees, or by employees, the self-employed or the non-employed on their own behalf) and they secure entitlement to health services.^2^ Consideration of these features led us to reclassify certain budget lines in the yearbooks of the National Health Insurance Fund Administration. For instance, in the yearbooks, item 8 (contribution for conscripts) was under the revenue category “social health insurance contributions and other contributions”; however, according to the 2011 version, the item is a tax transfer and not a health insurance contribution because it is paid from the government budget, i.e. it is not employment based, the first criterion of health insurance contributions.

**Table 2:** Revised revenue sources of the Hungarian health insurance fund for in-kind benefits (health services), 1995, 2000, 2005, 2008, 2010 and 2015

Revenue source	Adjustment made^a^	Item^b^	Revenue category^c^	Revenue in million Hungarian forints (% of total revenue) by year					
				1995	2000	2005	2008	2010	2015
**Social insurance contributions**	None	–	FS.3	275 887 (89.3)	324 353 (53.4)	645 544 (59.4)	689 358 (58.5)	431 812 (34.8)	405 164 (29.5)
Employer contribution^d^	Cash benefits revenue deducted	1	FS.3.2	181 653 (58.8)	263 209 (43.3)	478 814 (44.0)	382 160 (32.4)	119 791 (9.6)	–
Employee contribution^d^	Cash benefits revenue deducted	2	FS.3.1	29 705 (9.6)	57 591 (9.5)	160 312 (14.7)	298 508 (25.3)	287 890 (23.2)	372 676 (27.1)
Contribution by special groups, compulsory participation	None	3	FS.3.3	1 837 (0.6)	873 (0.1)	2 080 (0.2)	4 301 (0.4)	21 232 (1.7)	29 145 (2.1)
Contribution based on voluntary agreement	None	4	FS.3.3	–	510 (0.1)	650 (0.1)	226 (0.0)	229 (0.0)	333 (0.0)
Contribution paid in connection with short-term employment	None	6	FS.3.1	–	31 (0.0)	707 (0.1)	427 (0.0)	147 (0.0)	200 (0.0)
Late payment and other fines	Cash benefits revenue deducted	11	FS.3.4	5 966 (1.9)	2 139 (0.4)	2 981 (0.3)	3 736 (0.3)	2 523 (0.2)	2 810 (0.2)
Transfer from the pension insurance fund	None	21	FS.3.4	56 726 (18.4)	–	–	–	–	–
**Government domestic revenues**	None	–	FS.1	28 870 (9.3)	277 366 (45.6)	433 361 (39.9)	468 583 (39.7)	801 102 (64.5)	956 769 (69.6)
Employer social tax	Cash benefits revenue deducted	1	FS.1.2	–	–	–	–	–	247 717 (18.0)
Compensation by the labour market fund^e^	Cash benefits revenue deducted	7	FS.1.2	–	–	–	1 985 (0.2)	1 215 (0.1)	–
Contribution for conscripts	None	8	FS.1.2	–	560 (0.1)	–	–	–	–
Hypothecated health-care tax, lump-sum component	None	10	FS.1.2	–	169 152 (27.8)	138 091 (12.7)	85 890 (7.3)	7 040 (0.6)	–
Hypothecated health-care tax, proportional component	None	10	FS.1.2	–	12 227 (2.0)	26 317 (2.4)	33 078 (2.8)	34 167 (2.8)	166 362 (12.1)
Tax transfers for abortion; tax transfers for special health services	None	12; 14	FS.1.2	4 382 (1.4)	3 800 (0.6)	4 750 (0.4)	5 300 (0.4)	5 500 (0.4)	5 400 (0.4)
Tax transfers for non-contributing groups	None	13	FS.1.2	10 400 (3.4)	46 572 (7.7)	0	307 038 (26.0)	611 771 (49.2)	374 224 (27.2)
Tax transfer for unspecified tasks	None	17	FS.1.2	0	0	0	0	0	30 000 (2.2)
Mandatory payment of pharmaceutical companies	None	22	FS.1.2	–	–	–	35 292 (3.0)	38 265 (3.1)	54 981 (4.0)
Additional tax transfers from the 2007 surplus of the health insurance fund	None	26	FS.1.2	–	–	–	–	27 481 (2.2)	–
Tax on the premium of compulsory third-party liability insurance for motor vehicles	None	27	FS.1.2	–	–	–	–	–	27 493 (2.0)
Public health product tax on unhealthy food	None	28	FS.1.2	–	–	–	–	–	28 891 (2.1)
Tobacco industry health tax	None	29	FS.1.2	–	–	–	–	–	540 (0.0)
Tax transfer to cover the health insurance fund deficit	Cash benefits revenue deducted	A	FS.1.2	14 088 (4.6)	45 055 (7.4)	264 203 (24.3)	0	75 663 (6.1)	21 161 (1.5)
**Other revenues**	None	–	NA	4 030 (1.3)	6 051 (1.0)	8 276 (0.8)	21 048 (1.8)	9 342 (0.8)	11 883 (0.9)
User charges for abortion	None	18	FS.6.1	169 (0.1)	334 (0.1)	667 (0.1)	670 (0.1)	605 (0.0)	525 (0.0)
Reimbursement of health insurance fund expenditures on accidents and other injuries or damages^f^	None	19	FS.6.2	1 090 (0.4)	840 (0.1)	5 442 (0.5)	6 441 (0.5)	6 176 (0.5)	5 696 (0.4)
Reimbursement of health insurance fund expenditures based on international agreements^g^	None	23	FS.7.1	0	0	93 (0.0)	270 (0.0)	1 146 (0.1)	4 248 (0.3)
User charges for patient–doctor encounters and hospital stay, e.g. visit fee, hospital daily user charge	None	25	FS.6.1	–	–	–	10 960 (0.9)	–	–
Revenues from asset management	Cash benefits revenue deducted	30	FS.6.2	449 (0.1)	2 752 (0.5)	146 (0.0)	21 (0.0)	10 (0.0)	10 (0.0)
Revenues from administrative fees	Cash benefits revenue deducted	31	FS.6.1	2 322 (0.8)	2 125 (0.3)	1 928 (0.2)	2 686 (0.2)	1 404 (0.1)	1 404 (0.1)
**Total revenues (in-kind benefits)**	None	–	NA	308 787 (100.0)	607 770 (100.0)	1 087 181 (100.0)	1 178 989 (100.0)	1 242 255 (100.0)	1 373 816 (100.0)
**Deductions^h^**	None	–	NA	671 (NA)	1 175 (NA)	24 491 (NA)	5 066 (NA)	14 315 (NA)	12 327 (NA)
Other repayments and revenues	Cash benefits revenue deducted	20	NA	671 (NA)	694 (NA)	1 354 (NA)	1 224 (NA)	1 350 (NA)	998 (NA)
Repayment of pharmaceutical subsidies by pharmaceutical companies	None	22	NA	–	–	23 077 (NA)	3 507 (NA)	12 671 (NA)	10 292 (NA)
Repayment of health insurance fund reimbursements by health-care providers	None	24	NA	–	481 (NA)	60 (NA)	335 (NA)	294 (NA)	1 036 (NA)
**Cross-subsidy from cash-benefit revenues **	Expenditure side correction	B	NA	−32 924 (NA)	−51 461 (NA)	13 338 (NA)	−42 377 (NA)	−33 125 (NA)	−4 379 (NA)

FS: Classification of revenues of health financing schemes; NA: not applicable.

^a^ Revenues for cash benefits are not included in the figures because expenditures on cash benefits are not considered health expenditures, but social expenditures. Therefore, for revenue items that cover both cash and in-kind (health service) benefits, the cash benefits were deducted.

^b^ Numbers correspond to the numbering of items in Table 1. Letters are used for new items.

^c^ Revenue category refers to the classification of revenues of health-financing schemes according to the 2011 version of the System of Health Accounts.

^d^ The employee social health insurance contribution has a dedicated contribution rate for in-kind (health service) benefits and cash benefits, and the cash benefits were deducted accordingly. For example, in 2015, the employee social health insurance contribution was 7%, out of which 4/7 was for health services and 3/7 was for cash benefits. Therefore, from the total revenues, we deducted 42.9% (3/7) used to cover cash benefits, and the remaining 57.1% (4/7) used to cover in-kind (health service) benefits are included in the table. Where relevant, we used the same method to calculate the employer social health insurance contribution.

^e^ Compensation for the contribution relief in the Start Card programme and other tax transfers from the labour market fund.

^f^ Reimbursement by the responsible entity, e.g. compulsory third-party liability insurance for motor vehicles.

^g^ Such as European Union social security coordination and other bilateral agreements.

^h^ We excluded revenues that are the repayment of social health insurance payment to service providers, such as invalid payment claims, or subsidies above an agreed payment ceiling.

Notes: Dashes indicate that the revenue item had not yet been introduced or had been discontinued in that year. One million Hungarian forints are equivalent to 3700 United States dollars (2018 currency rate). The data are derived from the authors’ analysis based on Box 1, Table 1 and the Hungarian National Assembly acts,^22^^–^^27^ and using *A system of health accounts 2011*.^2^

We made the following adjustments to the data in Table 1. First, we excluded revenues for non-health expenditures, including cash benefits, such as sick pay (items 5, 9, 15 and 16 in Table 1), and deducted the cash-benefit parts of those revenue items, which are used for financing both cash benefits and health services at the same time (included in items 1 and 2 in Table 1, and the deficit of the health insurance fund, which is item A in Table 2). These deductions mostly explain why the totals in Table 1 are greater than those in Table 2.

Second, we analysed the revenue sources that did not come from taxes or health insurance contributions. Although these constitute a very small share of overall revenues of the health insurance fund, several items fall into these categories; for instance, user charges (items 18 and 25), other types of insurance (item 19), revenues from abroad (item 23) and revenues from asset management (item 30; Table 1).

Third, some items for taxes and health insurance contributions are misclassified. Most important are taxes on employers that do not match the definition of a social health insurance contribution since payment of these taxes does not provide entitlement to health insurance benefits. These include a hypothecated health-care tax (item 10) and the employer social tax,^29^ which replaced the employer social health insurance contribution in 2012 (item 1). The government removed the entitlement that came with the employer health insurance contribution to gain more control over allocation of these revenues and the decision on the benefits. Items 27, 28 and 29 are also an earmarked tax, with no entitlements, but these are not employment- or income-related revenues. Other items that required reclassification include the government’s Start Card programme, which aimed to support employment by covering part of the employer’s social health insurance contribution for people entering employment for the first time (item 7), and which was abolished in 2013, and the surplus of the health insurance fund in 2007 (item 26). Both items are also a domestic government revenue. In contrast, the 2011 version defines the transfer from the pension insurance fund to the health insurance fund to cover pensioners (item 21) as a special health insurance contribution revenue (FS.3.4); consequently, it belongs to the social insurance contribution category.^2^ The transfer from the pension insurance fund was abolished in 1997.^13^

Fourth, there are unusual revenue items, such as the repayment of National Health Insurance Fund Administration financing by health service providers for invalid payment claims (item 24) and the repayment of pharmaceutical subsidies by producers and distributors (item 22). We deducted these items from both the revenue and expenditure sides of the equation because they distort the actual spending figures by inflating them.

Fifth, the data published by the National Health Insurance Fund Administration do not include tax transfers made by the central government to cover the deficit of the health insurance fund (item A in Table 2). Similarly, we looked at the final expenditures on cash versus in-kind benefits and compared these figures with the total revenues calculated as described before. The balance is presented in Table 2 as item B (cross-subsidy from cash-benefit revenues). In years when total expenditures exceeded total revenues for health, we assumed that revenues for cash benefits were used to cover the difference (positive values of item B); in other years, the cross-subsidy worked the opposite way (negative values of item B).

**Fig. 2:**
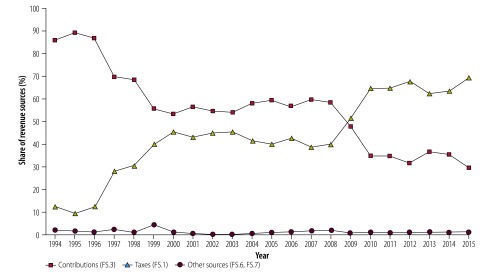
Changes in the share of revenue sources of the Hungarian health insurance fund, 1994–2015

Finally, we examined the changes in the revenue mix of the health insurance fund (Fig. 2) and the difference between the classifications of the 2000 and 2011 versions (Table 3). Although the tables show only selected years, Fig. 2 provides yearly data from 1994 to 2015.

FS: Classification of revenues of health financing schemes.

**Table 3:** Comparison of the mix of revenue sources of the Hungarian health system according the frameworks of the 2000 and 2011 versions of the System of Health Accounts, 2003, 2005, 2007 and 2009–2015

Indicator name in SHA version^2^^,^^3^	Revenue in million Hungarian forints (% of total revenue) by year									
	2003 (total revenue 1 553 519)	2005 (total revenue 1 804 098)	2007 (total revenue 1 854 429)	2009 (total revenue 1 915 388)	2010 (total revenue 2 047 250)	2011 (total revenue 2 135 031)	2012 (total revenue 2 148 834)	2013 (total revenue 2 195 782)	2014 (total revenue 2 311 636)	2015 (total revenue 2 444 117)
**Social health insurance contributions**										
2000 version: compulsory contributory health insurance	936 518 (60.3)	1 117 024 (61.9)	1 095 791 (59.1)	1 127 234 (58.9)	1 206 662 (58.9)	1 248 573 (58.5)	1 222 498 (56.9)	1 268 884 (57.8)	1 327 818 (57.4)	1 360 160 (55.7)
2011 version: social insurance contribution (FS.3)	500 327 (32.2)	668 430 (37.1)	651 086 (35.1)	535 847 (28.0)	414 665 (20.3)	431 979 (20.2)	390 643 (18.2)	458 001 (20.9)	471 323 (20.4)	400 933 (16.4)
**Government tax transfers**										
2000 version: general government	159 379 (10.3)	158 290 (8.8)	181 969 (9.8)	181 661 (9.5)	167 169 (8.2)	171 748 (8.0)	185 708 (8.6)	194 572 (8.9)	223 245 (9.7)	274 000 (11.2)
2011 version: transfers from government domestic revenues (FS.1)	586 855 (37.8)	597 287 (33.1)	606 006 (32.7)	766 266 (40.0)	949 327 (46.4)	981 775 (46.0)	1 002 668 (46.7)	995 821 (45.4)	1 071 040 (46.3)	1 223 557 (50.1)
**Voluntary contribution**										
2000 version: voluntary health insurance	9 553 (0.6)	20 344 (1.1)	39 123 (2.1)	53 324 (2.8)	57 820 (2.8)	57 073 (2.7)	59 034 (2.7)	60 070 (2.7)	59 162 (2.6)	56 257 (2.3)
2011 version: voluntary prepayment (FS.5)	9 553 (0.6)	20 344 (1.1)	39 123 (2.1)	53 324 (2.8)	57 820 (2.8)	57 073 (2.7)	59 034 (2.7)	60 070 (2.7)	59 162 (2.6)	56 257 (2.3)
**Out-of-pocket payments**										
2000 version: household out-of-pocket payments	409 433 (26.4)	464 816 (25.8)	486 996 (26.3)	502 494 (26.2)	561 273 (27.4)	602 428 (28.2)	631 149 (29.4)	622 733 (28.4)	655 085 (28.3)	705 799 (28.9)
2011 version: other domestic revenues from households (FS.6.1)	409 433 (26.4)	464 816 (25.8)	486 996 (26.3)	502 494 (26.2)	561 273 (27.4)	602 428 (28.2)	631 149 (29.4)	622 733 (28.4)	655 085 (28.3)	705 799 (28.9)
**Other**										
2000 version: other domestic private revenues (NPISH and enterprises)	38 636 (2.5)	43 625 (2.4)	50 550 (2.7)	50 676 (2.6)	54 326 (2.7)	55 209 (2.6)	50 446 (2.4)	49 521 (2.3)	46 326 (2.0)	47 901 (2.0)
2011 version: other domestic revenues from corporations and NPISH (FS.6.2, FS.6.3)	47 351 (3.0)	53 222 (3.0)	71 219 (3.8)	57 457 (3.0)	64 165 (3.1)	61 775 (2.9)	65 340 (3.0)	59 156 (2.7)	55 026 (2.4)	57 571 (2.4)

FS: Classification of revenues of health financing schemes; NPISH: non-profit institutions serving households; SHA: System of Health Accounts.

Note: Percentages do not always add up to 100% because of rounding errors.

## Changing mix of revenue sources

Based on the revisions we made using the 2011 version, Fig. 2 shows the changes in the mix of tax and contribution sources in the budget of the health insurance fund. What is apparent is that health insurance contributions are no longer the main source of health insurance revenues. This finding supports our argument that reporting of expenditures according to financing agent only, as done in the 2000 version, gave a misleading picture of the trends in health financing in Hungary. In Table 3, we compare the revenue structure of the entire Hungarian health system for selected years based on the classifications in the 2000 and 2011 versions of the System of Health Accounts. Using the 2000 version, all health expenditures of the National Health Insurance Fund Administration were classified as social security financing, and this accounted for 60.3% (936 518 million Hungarian forints/1 553 519 million Hungarian forints) of the total national health spending in 2003 and 55.7% (1 360 160 million Hungarian forints/2 444 117 million Hungarian forints) in 2015 (Table 3). Conversely, the classification of revenues of health-financing schemes used in the 2011 version breaks down social security financing into different revenue categories (FS.1–FS.7) and subcategories, of which only four (FS.3.1–FS.3.4) are considered social health insurance contributions. In 2015, social health insurance contributions accounted for only 16.4% (400 933 million Hungarian forints/2 444 117 million Hungarian forints) of the total resources of the whole health system (Table 3) and 29.5% (405 164 million Hungarian forints/1 373 816 million Hungarian forints) of the total revenues of the health insurance fund (Table 2). The rest of the health insurance fund revenues are mainly government domestic revenues (69.6%; 956 769 million Hungarian forints /1 373 816 million Hungarian forints; FS.1.2) and miscellaneous sources (0.9%; 11 883 million Hungarian forints/1 373 816 million Hungarian forints; FS.6 and FS.7; Table 2).

**Fig. 3:**
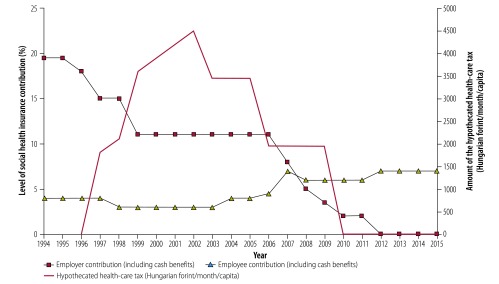
Changes in the social health insurance contribution rates of the Hungarian social health insurance system and the hypothecated health care tax, 1994–2015

Source: National Tax and Customs Administration^30^ and Hungarian National Assembly.^23^^,^^24^^,^^27^^,^^31^

Fig. 2 also shows that health insurance contributions fell sharply from 1995 to 1999 and then again from 2008 to 2010 when taxes became the main source of social health insurance revenues. In 2009, the share of tax revenues in the health insurance fund budget exceeded 50% (587 362 million Hungarian forints/1 133 039 million Hungarian forints) for the first time and reached over two thirds of the total revenues in 2015. Most of these changes can be explained if we follow the changes in health insurance contribution rates (Fig. 3). Over the years, the employer contribution rate has decreased substantially, from 19.5% in 1994 to 2% in 2011. The policy to reduce the employer contribution aimed to formalize the informal labour market and increase employment; policy-makers hoped that the lower health insurance contribution rates would encourage job creation and employment-generating investments in Hungary. The lost revenue to the health insurance fund has been partly compensated by the increase in the employee contribution rate for health services, from 3% in 2003 to 7% in 2007. Nevertheless, additional tax transfers from the central government budget were needed to fill the gap. Fig. 3 also shows that in 2012, the employer health insurance contribution decreased to 0%. This was not a further step to decrease the burden on employers. As mentioned earlier, the employer social insurance contribution was replaced by an earmarked social tax in 2012 at the same overall rate (27%) as in 2011. The overall rate includes contributions to pensions and unemployment benefits, not just social health insurance contributions. The employer social tax has no dedicated, fixed percentage part for health insurance and the actual revenue allocated from this source to the health insurance fund changes from one year to another. 

**Fig. 4:**
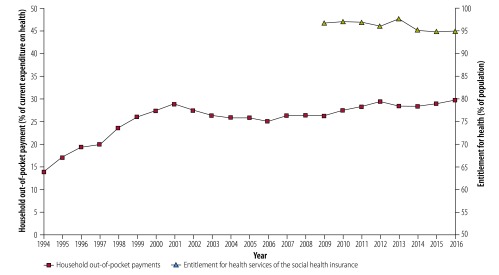
Changes in the share of out-of-pocket payments of the total expenditures for health of the Hungarian health system, 1994–2016, and the percentage of the population covered by the social health insurance scheme, 2009–2016

Notes: Entitlement data have only been available since 2009. Before 2007, entitlement to health services was not checked by the health service providers, so everybody who had a social insurance identification number could access care without restriction. The system to check entitlement was introduced in 2007, but during the first two years of operation, the entitlement database needed to be cleared of administrative errors. Out-of-pocket payments include user charges in the social health insurance system and payments for private health services and goods, which are not covered by the social health insurance system. It is not possible to separate the two and therefore the out-of-pocket data presented here are for the whole Hungarian health system.

Source: Hungarian Central Statistical Office^14^ and World Health Organization.^12^

We also looked at whether the increased share of central government tax financing influenced population coverage and the level of out-of-pocket payments. Fig. 4 shows that there was no improvement in either measure of coverage.

## Discussion

The finding that Hungary has a hybrid tax-funded social health insurance system suggests that the traditional models to describe health-financing systems, such as the tax-based Beveridge-type national health system or the contribution-based Bismarck-type social health insurance system, are not adequate for exploring policy options to improve performance. Indeed, it was the growing recognition that mixed models are common, with evidence of countries channelling general budget revenues to a purchasing agency rather than directly to the supply side,^32^ that led to the separate classification of revenue sources and financing schemes in the 2011 version of the System of Health Accounts. To accurately document and analyse how countries have adapted their revenue mix to changing macroeconomic circumstances will require international reporting of health expenditures according to the two classifications in the 2011 version: revenues of health-financing schemes and health-financing schemes.

The case of Hungary also illustrates that mixing features of revenue sources of the classic Bismarck and Beveridge models into a hybrid tax-funded social health insurance system might be one way to sustain health system performance in a fast-changing social and economic environment.^33^^–^^35^ Proposals for increasing the share of general tax revenues in health financing are based on the argument that taxes are a more stable funding source, are less affected by fluctuations in economic cycles, and spread the tax burden over a wider population base. These features of tax financing are important for sustainability in higher-income countries with ageing populations because the relative size of the working-age population is shrinking. Thus, relying solely on wage-based contributions would require raising rates, with harmful consequences for employment and economic competitiveness.^5^^,^^6^^,^^36^ These considerations are also relevant to lower-income countries with large informal economies which have, or are considering, social health insurance because the revenues that can be generated from wage-linked contributions are limited and thus they must depend on government budgets to expand coverage.^37^ In each case, sustained progress towards universal health coverage (UHC) in social health insurance systems would not be possible without additional tax financing. While progress towards UHC is best served where health systems rely predominantly on public revenue sources, factors such as economic growth, tax policy and labour market considerations mainly determine the mix of such sources.^37^ These factors are outside the influence of the health sector and are distinct from health-policy choices regarding pooling and purchasing arrangements. Hungary provides an example of moving away from traditional contribution-dominated revenue sources to a greater reliance on general taxation. The country is now at the point where revenues from taxes to fund the social health insurance system well exceed revenues from health insurance contributions. We argue that for an optimal combination of revenue sources, which provides long-term sustainability and greater potential for progress towards UHC, all options available need to be considered, which contrasts with the simplistic choice between Beveridge or Bismarck models. The theoretical basis for this potential paradigm shift lies in the functional analysis and deconstruction of health systems.^38^^–^^41^

The Hungarian case has other implications. The increasing reliance on tax revenues created the opportunity to tackle gaps in population coverage by changing the basis of entitlement, and to increase financial protection by decreasing out-of-pocket payments. This change, however, did not happen. The finding that increasing government transfers does not necessarily lead to coverage expansion highlights the importance of governance. In terms of revenues, the Hungarian government focused on decreasing the presumed adverse effect of health insurance contributions on the labour market and failed to recognize the broadening health policy options that came with tax financing. In this respect, the case of Hungary is an example of missed opportunity because of the lack of a consistent and transparent health-financing strategy based on policy objectives shared and agreed between the health and financial decision-makers.

The example of Hungary shows that an accurate analysis of health expenditure data that provides a detailed description of the revenue sources for public expenditure on health is essential for sound, evidence-based policy-making. Without analysing the health financing system accurately, we would fail to see that Hungary has moved towards a tax-funded social health insurance model. Furthermore, not tracking revenue sources accurately might have been one of the reasons why the government did not recognize the broadening policy options to tackle coverage gaps. Once adopted for international reporting by all countries, the 2011 version of the System of Health Accounts will facilitate improved international comparison of revenue sources for health.
